# Evaluation of the Distribution of *Candida Species* in Patients with Dysplastic and Nondysplastic Oral Lichen Planus Lesions

**DOI:** 10.1155/2022/8100352

**Published:** 2022-06-01

**Authors:** Fahimeh Rezazadeh, Morteza Beirami, Zahra Zareshahrabadi, Hossein Sedarat, Kamiar Zomorodian

**Affiliations:** ^1^Oral and Dental Disease Research Center, Department of Oral and Maxillofacial Medicine, School of Dentistry, Shiraz University of Medical Sciences, Shiraz, Iran; ^2^Student Research Committee, School of Dentistry, Shiraz University of Medical Sciences, Shiraz, Iran; ^3^Basic Sciences in Infectious Diseases Research Center, Shiraz University of Medical Sciences, Shiraz, Iran; ^4^Student Research Committee, Jahrom University of Medical Sciences, Jahrom, Iran; ^5^Department of Medical Mycology and Parasitology, School of Medicine, Shiraz University of Medical Sciences, Shiraz, Iran

## Abstract

**Objectives:**

This study is aimed at identifying and determining the distribution of isolated *Candida* species in patients with dysplastic and nondysplastic oral lichen planus (OLP) lesions in comparison with those of healthy controls. *Material and Methods*. This study includes patients with OLP, aged (more than 18 years old), who have had informed consent. Samples of the oral, tongue, and buccal mucus by rubbing with a sterile swab and sterilely next to the lamp flame. Demographic information was obtained using patient records to determine the species of *Candida* in both groups, and two tests of fertile tube production by *Candida albicans* and dye production in the dye medium were used. A biopsy from OLP lesions has been taken from each patient after swab sampling and was sent to the pathology department for further histopathological analysis. In the end *p* value, less than 0/05 was considered significant.

**Result:**

In this study, 40 lichen planus patients were compared with 32 control patients. The female/male ratio in OLP and healthy groups was 22/18 and 17/15, respectively. Among the OLP patients, 23 cases (56%) were dysplastic, and the other 17 (44%) patients were nondysplastic. The mean (±standard deviation (SD)) age of patients was 48.83 (±9.34) years, and the mean age of the control group was 40.21 (±10.32). There were no significant differences based on age (*p* > 0.05). The highest frequency was related to tongue in both groups (22 (55%)) and buccal mucosa was the least common. There was a significant relationship between the location of the lesion and OLP (*p* = 0.05). 18 (45%) were erosive, and 22 (55%) were nonerosive. However, no significant difference was observed between erosive and nonerosive types in the OLP group (*p* = 0/07). Regarding the type of *Candida*, all cases in the patient's group were related to *Candida albicans* [40 (100%)], and the correlation was not found in this regard (*p* > 0/05). About colony count, the mean for the case and control groups was 26.68 and 23.25, respectively. Also, no significant relationship was found between colony count and groups in this study (*p* = 0.3). There was no significant difference between gender and dysplastic or nondysplastic (*p* > 0.05).

**Conclusion:**

According to the statistical studies performed in this study, the presence of Candida in patients with dysplastic and nondysplastic lichen planus is not significantly different, and this rate is not higher than healthy individuals and in cases where the results are positive. The predominant species of Candida is the Candida albicans. In this study, the highest frequency was related to tongue in both groups. There was only a significant relationship between the location of the lesion and OLP.

## 1. Introduction

Oral lichen planus (OLP) is a refractory and chronic inflammatory disease [1]. This autoimmune disorder is usually difficult to manage due to its unknown cause [2]. It seems to be a T-cell mediated disorder in which they target intrinsic and extrinsic cell antigens. Genetics, trauma, stress, and infection may also play a part in OLP. Reticular, popular, plaque-like, and erosive/ulcerative are different types of OLP [3]. The first three are usually symptom free and just seen with white striations, papules, or plaques. Atrophic/erosive types may cause discomfort, ulceration, and burning sensation [4]. They represent a diffuse, red, atrophy, or erosion with white striations around them [5]. OLP lesions often present in a background of erythema which is commonly infected with candida [6]. OLP usually persists for years and may undergo malignant transformation or spontaneous remission [1]. World Health Organization considers this disease as a precancerous lesion [2]. The rate of malignancy in OLP is not very high and varies from 0.3% to 3% and is more frequently in erosive/atrophic and plaque forms. Because of this tendency, patients with OLP usually need to follow periodic. There is no specific treatment for OLP but surgical (cryosurgery and carbon dioxide laser ablation) and nonsurgical (topical, intralesional or systemic corticosteroids, retinoid, and cyclosporine) measures are taken by the clinicians [5]. Chlorhexidine mouthwashes have good effects on some lesions, indicating that microbiota may have a role in OLP exacerbations [6]. Furthermore, antifungal therapy results in regression of OLP type, meaning that *Candida albicans* (*C. albicans*) may have the same effect. *Candida* albicans is an opportunistic fungal pathogen found as part of the normal microflora in the human digestive tract which may become pathogenic when the immune defense system of that host is weakened. The relationship between OLP and candidiasis is still unclear [7]. *C. albicans* may play a role in OLP dysplasia by producing carcinogenic compounds like nitrosamines and N-nitrosobenzylmethylamine. Researches were shown that strains isolated from more dysplastic lesions have more potency in nitrosation which means C. albicans may play a key role in the development of dysplasia [7]. The prevalence of *Candida* species in OLP patients compared to healthy individuals is also controversial [3]. Masaki et al. in 2011 have worked on the detection and identification of non-Candida albicans species in human oral lichen planus and concluded that Candida colonization is more likely detected in subjects with OLP. Non-*C. albicans* species are specifically detected in OLP patients, particularly those with OLP and diabetesis [8]. Artico et al. in 2014 have worked on the prevalence of *Candida* spp., xerostomia, and hyposalivation in oral lichen planus and concluded that a higher prevalence for *Candida* colonization was found in healthy people in comparison to OLP patients and patients with oral lesions other than OLP which is in contrast with the above study. No statistical difference was found regarding colonization of Candida spp. in the reticular and atrophic forms of OLP [1]. Mehdipour et al. in 2010 have worked on prevalence of *C. albicans* in erosive OLP and found no difference between healthy subjects and those with erosive OLP [2]. Generally with regard to variety of *Candida* species susceptibility to antifungal drugs, and patients with oral lichen planus with different disease manifestations usually have Candida infection, it is logical to correctly identify *Candida* species to start antifungal treatment.

Obviously, the prevalence and superimposition of *Candida* species on different types of OLP and its effect on OLP dysplastic changes are controversial. Regarding the lack of sufficient studies about the risk factors of dysplasia occurrence in OLP; the aim of this study is to identify and determine the distribution of isolated *Candida* species in patients with dysplastic and nondysplastic OLP lesions in comparison with those of healthy controls.

## 2. Materials and Methods

This cross-sectional study included 40 patients with OLP and 32 healthy persons, (aged more than 18 years old), who were referred to the Department of Oral medicine of Shiraz Dental School. This project has been approved by the ethics committee of Shiraz University of Medical Sciences (IR.SUMS.DENTAL.REC.1400.008).

**Figure 1:**
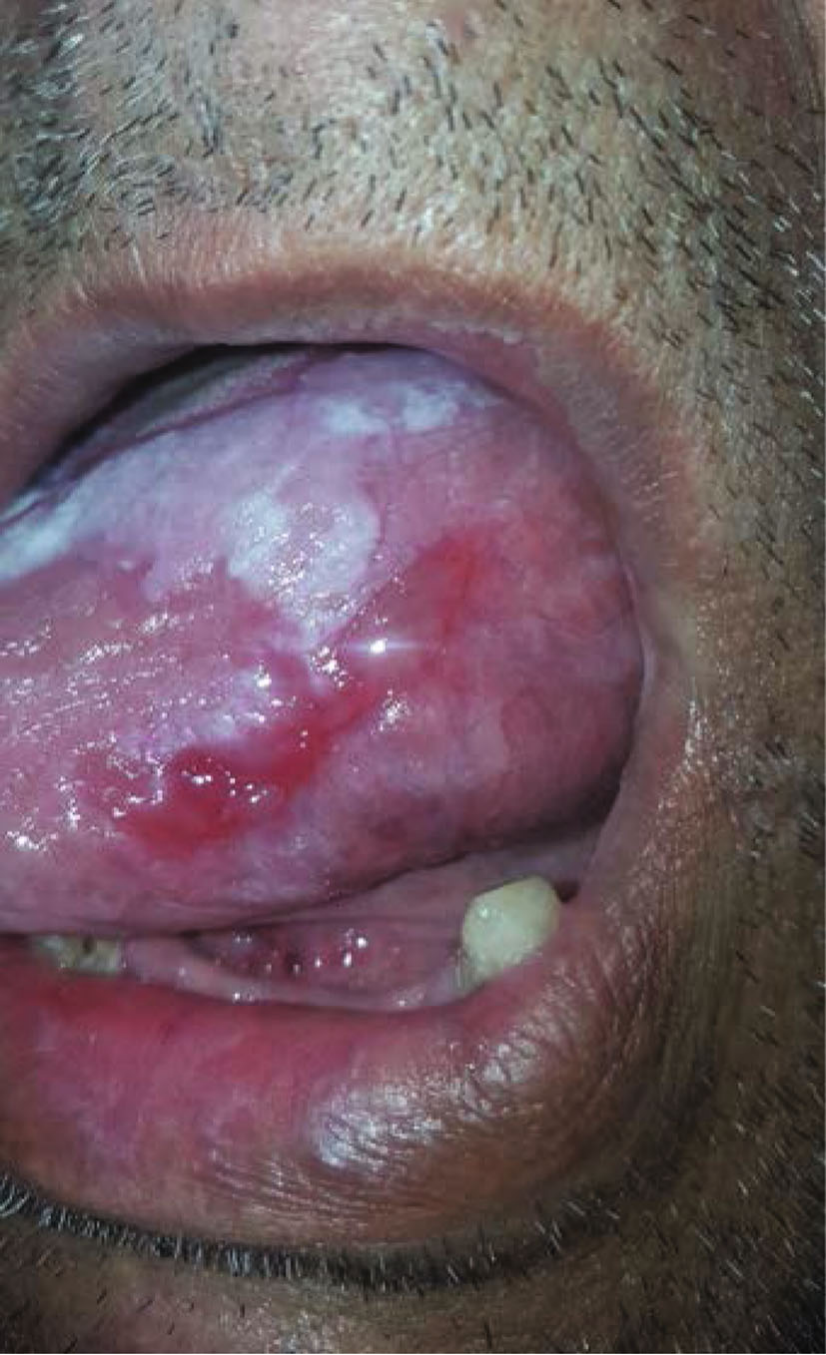
Dysplastic OLP lesion.

In this study, three groups of patients were enrolled:

*Group 1*: the OLP group with dysplasia (*n* = 23) (Figure 1)

**Figure 2:**
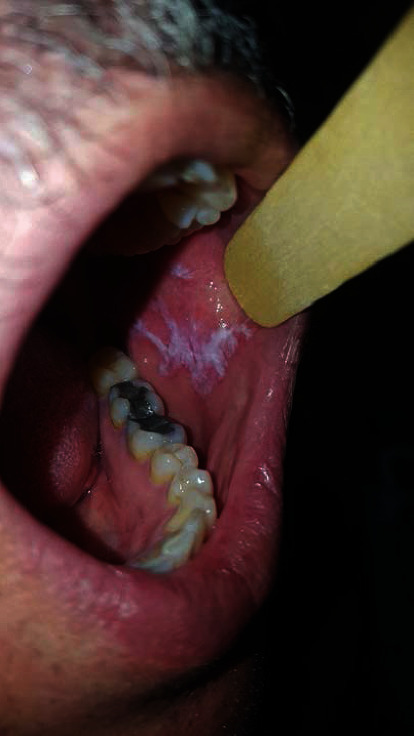
Nondysplastic OLP lesion.

*Group 2*: the OLP group without dysplasia (*n* = 17) (Figure 2)

*Group 3*: the healthy subjects (*n* = 32)

OLP lesions were diagnosed based on clinical and pathological methods with the clinical forms of the disease categorized as (erosive/nonerosive).

Patients with these criteria were excluded from the study.

(a) The existence of any predisposing factor for candidiasis such as
  1. Long-term use of antibiotics and steroid therapy (systemic or local)
  2. Congenital or acquired defects predisposing to candidiasis such as diabetes mellitus, AIDS, chemotherapy, and addiction
  3. Congenital syndromes such as DiGeorge, cutaneous-mucosal candidiasis
  4. People who smoked
(b) The presence of any cause of lichenoid reactions
(c) People under 18 years of age or over 60 years
(d) Receiving any antifungal or topical steroid treatments during the last 1 month
(e) The patient's unwillingness to participate in this study

A questionnaire was prepared for all patients including gender, type of OLP, dysplastic changes, biopsy site, duration of disease, lesion site, swab sample site, history of recurrency, previous treatments for OLP, and underlying diseases.

*Candida* colony count evaluation was obtained by applying a sterile swab on the affected mucosa, before a biopsy. Thereafter, the swab was placed in a sterile test tube containing 5 ml of sterile phosphate-buffered saline and vortexed gently to detach the organisms from the swab.

**Figure 3:**
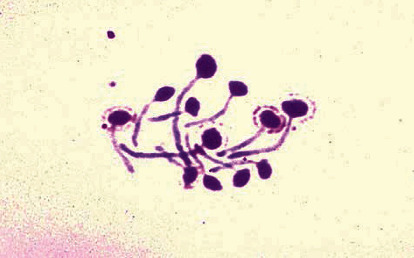
Production of germinal tubes by *Candida albicans.*

**Figure 4:**
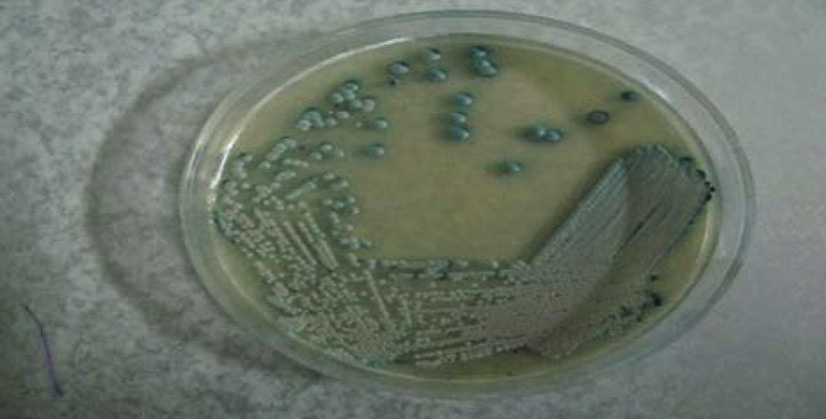
Cultivation of *Candida albicans* in chromium agar medium.

Aliquots of the suspension (50 *μ*l) were directly spread on CHROM agar Candida medium ((HiMedia, Mumbai, India)) and sabouraud dextrose agar (SDA) (Merck, Germany) containing chloramphenicol to identify *Candida species* and quantification of colonies, respectively. Then, plates are incubated at 32C for 24-48?h, and the number of colonies on each plate was counted (CFU/mL). To determine the species of *Candida* in both groups, two tests of fertile tube production by *C. albicans* and dye production in the CHROM agar Candida medium were used (Figures 3 and 4). The presence of *C. albicans* was confirmed by the typical green colonies grown on the mentioned media.

A biopsy from OLP lesions had been taken from each patient after swab sampling and was sent to the pathology department for further histopathological analysis to determine dysplastic change.

## 3. Statistical Analysis

Finally, results were analyzed with SPSS program v.24. Statistical tests including chi-square for assessing the relation between sex, dysplastic change and Candida species, *t*-test for assessing the relation between age, CFU and Candida species, odds ratio, and the Mann–Whitney test for analysis nonparametric data. *p* value less than 0/05 was considered significant.

## 4. Result

In this study, a total of 72 samples were examined. 40 lichen planus patients were compared with 32 control patients. The female/male ratio in OLP and healthy groups was 22/18 and 17/15, respectively. Among the OLP patients, 23 cases (56%) were dysplastic, and the other 17 (44%) patients were nondysplastic.

The mean (±standard deviation [SD]) age of patients was 48.83 (±9.34) years, and the mean age of the control group was 40.21 (±10.32). The results of the *t*-test for equality of means age were not significant (*p* > 0.05) so the two groups were not significant differences based on age.

### 4.1. Frequency of Patients according to the Location of the Lesion

The following information was obtained about the location of the lesion sampling in patients. The highest frequency was related to tongue in both groups [22 (55%)], and buccal mucosa was the least common. There was a significant relationship between the location of the lesion and OLP (*p* = 0.05). Other information about the location of the lesion is mentioned in the table below (Table 1).

### 4.2. Frequency of Erosive and Nonerosive in Lichen Planus Patients

Of the 40 lichen samples studied, 18 (45%) were erosive, and 22 (55%) were nonerosive. However, no significant difference was observed between erosive and nonerosive types in the OLP group (*p* = 0/07).

### 4.3. Frequency of *Candida* Type in the OLP Patient and Control Group

Regarding the type of *Candida*, all cases in the patient's group were related to *Candida albicans* [40 (100%)]. Concerning the control group, the highest frequency was related to *Candida albicans* (92%). In the control group, only 2 cases were not *C. albicans* in these subjects of which one was *C. glabrata* and one was *C. parapsilosis*.

### 4.4. Colony Production and Count in the OLP Patient and Control Group

In this study, in both control and lichen planus groups, all samples were able to produce fertile tubes with a positive culture, except for two cases that were observed in healthy individuals.

About colony count, the mean for the case and control groups was 26.68 and 23.25, respectively. Also, no significant relationship was found between colony count and groups in this study (*p* = 0.3) (Table 2).

### 4.5. The Relationship between Gender, Age, and OLP

There was no significant relationship between gender, age, and lichen plan (*p* > 0.05).

### 4.6. The Relationship between Gender and Being Dysplastic or Nondysplastic

In the female population of OLP group (*n* = 22), 14 (63.6%) were dysplastic, and 8 (36.3%) were nondysplastic. Also, in the male population (*n* = 18), 8 (44.4%) were dysplastic, and 10 (55.5%) were nondysplastic. However, there was no significant difference between gender and dysplastic or nondysplastic (*p* > 0.05) (Table 3).

## 5. Discussion

The present study determined the presence of *Candida species* in patients with dysplastic and nondysplastic lichen planus and compared the results with healthy individuals. In this study, 40 lichen planus patients were compared with 32 control patients. There was no significant relationship between age and lichen plan which is in line with study related to candidiasis and lichen planus of patients [9].

In our study, there was no significant difference between gender and dysplastic or nondysplastic. In this regard, it is in line with the study of Susan et al. which indicates the lack of association between lichen planus disease and the presence of *Candida albicans* and gender, and stated that in this context it is logical that no significant relationship is observed because factors such as oral health and sometimes genetics and place of residence can be related. Be with *Candida* and lichen planus [10].

The highest number in this study was related to *Candida albicans*. It seems that *Candida albicans species* has the highest association with lichen planus compared to other species. This is probably due to the greater ability of this type of candidate to cause more pathogenicity in the mouth [11].

In the study of samples transferred to the laboratory by the direct method, the presence of a high normal number of yeast cells or with pseudohyphae indicating colonization of the fungus and candidiasis was considered. Examination of the results of this experiment showed that the difference between *Candida* infection in the healthy group and with erosive lichen planus lesions is statistically not significant. These results suggest that no significance in this regard probably due to the low number of patient samples, especially dysplastic patients in this study. Singh et al.'s study revealed a statistically nonsignificant correlation between the presence of *Candida* and epithelial dysplasia in oral mucosal lesions. In this researcher's study, a low sample size and 50 patients with lichen planus were studied [12].

The following information was obtained about the location of the lesion sampling in patients. The highest frequency was related to tongue in both groups, and buccal mucosa was the least common. There was a significant relationship between the location of the lesion and OLP. Based on Singh et al.'s study, this correlation may also be possible that the increased colonization and prevalence of oral yeast in these lesions are entirely coincidental and merely reflects a changing local environment that allows for the proliferation of these common oral commensals. Thus, the presence of *Candida* itself may not be sensitive enough to draw any definite conclusion regarding malignant potential [3].

In our study, in both control and lichen planus groups, all samples were able to produce fertile tubes with a positive culture, except for two cases that were observed in healthy individuals. About colony production, no significant relationship was found. The difference between the study biopsy result and the prepared smear can be explained by the *Candida* colonization occurs at the lesion surface, and *Candida* invasion of the tissue is observed when the tissue has very low resistance. Therefore, it is better to check the presence of *Candida* with potash in samples that were positively cultured for further confirmation [13].

In both dysplastic and nondysplastic cases, there was no significant correlation between colony formation and OPL. Many studies deal with this matter, for example, Sarkar and Rathod in their study have shown that forty percent of leukoplakia cases were simultaneously positive for *Candida* on direct microscopy, culture, and histopathologic evaluation. No significant difference was found between nondysplastic and distinctly dysplastic lesions concerning *Candida* detection on microscopy or culture [14].

The results of this study in comparison with the studies conducted by Roy et al. and He et al. also confirm this. However, these two researchers examined the samples in two ways. In one case, they examined the presence of hypha by potash staining, and in the second case, they examined the invasion of *Candida* in the biopsy samples of patients [15, 16]. He et al. in 1 of 43 biopsies and Krogh in none of the 19 samples showed *Candida* invasion. He et al. reported 10 positive samples with potash staining and Krogh 37% of the samples positive [16].

In the study, Irani et al. examined the prevalence of *Candida* in patients with lichen planus. Out of 185 patients with biopsy, 34% had Candida infection, while out of 120 healthy individuals, only two cases of *Candida* infection were reported [10].

The difference between the results of a study by Irani et al. and this study can be due to the large number of samples examined and the method of examining the prepared samples which were biopsies.

Diagnosis of dysplastic lesions and manage them is an important topic especially in OLP patients. So the effect of *Candida species* on dysplasia can be vital in the treatment of these group [17, 18].

In the present study, only 2 samples prepared from healthy individuals were negative in fertile tube production and the rest of the samples all confirmed the presence of albicans *Candida* in positively cultured individuals.

This result confirms previous studies that have all pointed to the high prevalence of *Candida* albicans in healthy individuals and patients with lichen planus.

In 2014, Shukla et al. examined the *Candida species* common in patients with lichen planus and declared *albicans* the predominant *Candida species*, although it had isolated other groups of *Candida* in patients [9].

Many factors are involved in increasing the colonization of this fungus, including immunosuppression, medications, malnutrition, malignancy, age, and poor oral hygiene. In this study, no significant differences were found regarding the presence of dysplastic or nondysplastic individuals and the presence of *Candida species* [19].

Regarding the limitation of our study due to patients' enrollment in coronavirus pandemic, it is important to mention that the sample size of the present study was low, and for a more detailed study, more studies with a higher sample size are needed.

Suggestions:

1. Doing a study with a larger sample size for a more detailed study of this important
2. Evaluation of other *Candida albicans species*. A more general and accurate comparison in this regard
3. Carrying out purposeful studies concerning the better understanding of the factors related to oral lichen planus

## 6. Conclusion

According to the statistical studies performed in this study, the presence of *Candida* in patients with dysplastic and nondysplastic lichen planus is not significantly different, and this rate is not higher than healthy individuals. The predominant type of *Candida* is the *albicans* species. In this study, the highest frequency was associated to tongue in both groups. There was only a significant relationship between the location of the lesion and OLP.

## Figures and Tables

**Table 1:** Frequency of patients according to the location of the lesion.

Location	OLP patients	*p* value
Buccal mucosa		
Count	8	0.05
Percent	24.2%	
Gingiva		
Count	10	
Percent	14.0%	
Tongue		
Count	22	
Percent	13%	
Total		
Count	40	

**Table 2:** Mean of colony count in lichen planus (case) and healthy (control) group.

	Number	Mean rank of colony count	*p* value
Group			
Case	40	26.68	0.3
Control	32	23.25	

**Table 3:** The relationship between gender and dysplastic and being nondysplastic.

	Number (percent)	*p* value _intergroup_
Sex		
Female		
Dysplastic	14 (63%)	0.31
Nondysplastic	8 (36%)	
Male		
Dysplastic	8 (44%)	0.82
Non-dysplastic	10 (55%)	

## Data Availability

Data of the participant can be requested from the authors. Please write to the corresponding author if you are interested in such data.
